# Efficacy and safety of apatinib plus immune checkpoint inhibitors and transarterial chemoembolization for the treatment of advanced hepatocellular carcinoma

**DOI:** 10.1007/s00432-024-05854-8

**Published:** 2024-07-08

**Authors:** Jianfei Wu, Xuefeng Bai, Guodong Yu, Quan Zhang, Xixi Tian, Yuan Wang

**Affiliations:** Department of Hepatobiliary Surgery, Affiliated Hospital of Hebei University, Baoding, 07100 China

**Keywords:** Apatinib, Immune checkpoint inhibitors, Transarterial chemoembolization, Advanced hepatocellular carcinoma, Efficacy and safety

## Abstract

**Purpose:**

The evidence of apatinib plus immune checkpoint inhibitors (ICIs) and transarterial chemoembolization (TACE) for treating advanced hepatocellular carcinoma (HCC) is limited. This study aimed to compare the treatment efficacy and safety of apatinib plus ICIs and TACE with apatinib plus TACE in these patients.

**Methods:**

This study retrospectively enrolled 90 patients with advanced HCC treated with apatinib plus TACE (A-TACE group, *n* = 52) or apatinib plus ICIs and TACE (IA-TACE group, *n* = 38).

**Results:**

The objective response rate was numerically higher in IA-TACE group compared with A-TACE group without statistical significance (57.9% vs. 36.5%, *P* = 0.055). Disease control rate was not different between groups (86.8% vs. 76.9%, *P* = 0.248). Progression-free survival (PFS) was improved in IA-TACE group compared with A-TACE group (*P* = 0.018). The median PFS (95% confidence interval) was 12.5 (8.7–16.3) months in IA-TACE group and 8.5 (5.6–11.4) months in A-TACE group. Overall survival (OS) was also prolonged in IA-TACE group compared with A-TACE group (*P* = 0.007). The median OS (95% confidence interval) was 21.1 (15.8–26.4) months in IA-TACE group and 14.3 (11.5–17.1) months in A-TACE group. By multivariate Cox regression model, IA-TACE was independently associated with prolonged PFS (hazard ratio = 0.539, *P* = 0.038) and OS (hazard ratio = 0.447, *P* = 0.025). Most adverse events were not different between groups. Only the incidence of reactive cutaneous capillary endothelial proliferation was higher in IA-TACE group compared with A-TACE group (10.5% vs. 0.0%, *P* = 0.029).

**Conclusion:**

Apatinib plus ICIs and TACE may be an effective and safe treatment for patients with advanced HCC, but further large-scale studies are needed for verification.

**Supplementary Information:**

The online version contains supplementary material available at 10.1007/s00432-024-05854-8.

## Introduction

Hepatocellular carcinoma (HCC) is one of the most prevalent and lethal cancers worldwide (Brown et al. 2023; Singal et al. 2023). In China, the incidence and mortality of HCC are approximately 410 and 391 per 100,000 population (Cao et al. 2021). Advanced HCC refers to the stage with portal vein invasion and/or extrahepatic disease, well-preserved liver function, and good physical performance (Vogel et al. 2022). For patients with advanced HCC, the recommended standard treatment according to the guidelines in China is transarterial chemoembolization (TACE) with or without systematic therapy, which includes tyrosine kinase inhibitors (TKIs) (such as sorafenib, lenvatinib, and donafenil), chemotherapy based on oxaliplatin, and atezolizumab plus bevacizumab (Chen et al. 2020a, b; Xie et al. 2023a, b). However, the outcomes of these patients are still not satisfactory (Chen et al. 2022a, b; Wang et al. 2022; Zanuso et al. 2023).

Apatinib is an oral-administrated TKI that selectively inhibits vascular endothelial growth factor (VEGF) receptor 2 to exert anti-tumor effects (Li et al. 2022a, b). Apatinib is originally served as a therapeutic agent for gastric cancer, while recent studies have revealed that it also shows potential for treating other cancers including non-small cell lung cancer, breast cancer, and gastric cancer (Geng and Li 2015; Xue et al. 2018; Liu et al. 2020a; Li et al. 2022a, b). In HCC, apatinib is regarded as the second-line systemic treatment, and its combination with TACE is also recommended for the treatment of advanced HCC (Xie et al. 2023a, b). Immune checkpoint inhibitors (ICIs), such as atezolizumab, camrelizumab, and sintilimab, are able to activate the anti-tumor immune response and thus serving as a treatment modality for advanced cancers (Cheu and Wong 2021; Harkus et al. 2022). Interestingly, apatinib ICI could improve the survival of patients with HCC (Yuan et al. 2020; Xu et al. 2021; Chen et al. 2022a, b; Li et al. 2022a, b). It is also reported that TACE induces immunogenic tumor cell death in patients with HCC, which provides the theoretic basis for combining TACE with ICIs (Yuan et al. 2020). Based on these information, it could be speculated that apatinib combined with TACE and ICIs may serve as a potential treatment for advanced HCC. However, only three studies have explored this issue (Liu et al. 2023; Sun et al. 2023; Xia et al. 2023). Therefore, more evidence is needed to further verify the treatment potential of apatinib combined with TACE and ICIs for advanced HCC and to facilitate its clinical application.

The current study intended to compare the treatment response, survival, and adverse events in patients with advanced HCC who received apatinib combined with TACE and ICIs and those who received apatinib combined with TACE.

## Methods

### Patients

This study retrospectively screened 90 advanced HCC patients treated with apatinib combined with TACE (A-TACE, as A-TACE group) or ICIs plus A-TACE (IA-TACE, as IA-TACE group) from May 2020 to July 2023. The inclusion criteria were: (i) firstly diagnosed as HCC by the American Association for the Study of Liver Diseases (Marrero et al. 2018); (ii) adult patients (≥ 18 years old); (iii) Child-Pugh stage of A or B; (iv) Eastern Cooperative Oncology Group performance status (ECOG PS) of 0 to 1; (v) Barcelona Clinic Liver Cancer (BCLC) stage of C; (vi) received at least one cycle of IA-TACE or A-TACE treatment; (vii) had at least one available data of best response by Modified Response Evaluation Criteria in Solid Tumors (mRECIST) guideline (Lencioni and Llovet 2010). The exclusion criteria were: (i) had a history of other cancer; (ii) received previous cancer treatment; (iii) had missing follow-up data. This study gained approval from the Ethics Committee. All patients, or their families, have given their informed consent.

### Data collection

Clinically relevant characteristics were collected, which contained age, sex, hepatitis B virus (HBV), liver cirrhosis, ECOG PS, Child-Pugh stage, maximum tumor diameter, portal vein invasion, extrahepatic disease, BCLC stage, China liver cancer (CNLC) stage, alpha-fetoprotein (AFP), albumin (ALB), total bilirubin (TBIL), alanine aminotransferase (ALT), aspartate aminotransferase (AST), alkaline phosphatase (ALP), programmed cell death ligand 1 combined positive score (PD-L1 CPS), and times of TACE.

Treatment information was screened, and the conventional treatment regimen was as follows: within 7 days after traditional TACE, apatinib combined with or without ICI was initiated. ICI type contained camrelizumab, sintilimab, atezolizumab, and tislelizumab. The dosage of apatinib was 250 mg/day until intolerance or disease progression, and the dose could be reduced to 250 mg/2 days depending on the patient’s disease or physical condition. The dosage of ICI was as follows: 200 mg/cycle for programmed cell death-1 (PD-1) inhibitors, and 1200 mg/cycle for PD-L1 inhibitors, both with a 21-day cycle until intolerance or disease progression.

Clinical response data were collected, which was evaluated at the 2/4/6/8 cycles after conventional treatment initiation, followed by evaluations every 3 months. The best response was screened for analysis. Besides, the disease progression or death conditions were collected to calculate accumulating progression-free survival (PFS) or overall survival (OS) rates. Adverse events were also collected.

### Data analysis

Continuous characteristics were compared using the Mann-Whitney U test or independent-sample T-test, and categorical characteristics were compared via the *χ*^*2*^ test or Fisher’s exact test between groups. The accumulating PFS rate and accumulating OS rate were graphed by the Kaplan-Meier method, in which the Log-rank test was utilized to compare those rates between groups. To identify factors related to PFS and OS, univariate and backward-multivariate Cox regression models were conducted. The univariate Cox regression model was also used to compare PFS or OS between patients who received IA-TACE and A-TACE in different subgroups. Data analyses were performed by SPSS v.26.0 (IBM, America). Statistical significance was considered to be achieved when the *P* < 0.05 (two-sided).

## Results

### Baseline characteristics

IA-TACE group had a median (interquartile range (IQR)) age of 56.0 (51.8–63.8) years, consisting of 34 (89.5%) males. A-TACE group had a median (IQR) age of 54.0 (48.3–62.0) years, containing 43 (82.7%) males. Comparison analyses revealed that the proportion of patients with abnormal TBIL was higher in IA-TACE group compared with A-TACE group (40.5% vs. 13.5%, *P* = 0.004). While the other characteristics were not different between groups, including age, male, HBV, liver cirrhosis, ECOG PS, Child-Pugh stage, tumor characteristics, disease stage, liver function indexes, and times of TACE (all *P* > 0.05). The detailed baseline characteristics of the two groups are shown in Table 1.

**Table 1:** Characteristics of advanced HCC patients

Characteristics	All (*N* = 90)	IA-TACE (*n* = 38)	A-TACE (*n* = 52)	*P* value
Age (years)	55.0 (49.8–63.0)	56.0 (51.8–63.8)	54.0 (48.3–62.0)	0.377
Male sex	77 (85.6)	34 (89.5)	43 (82.7)	0.366
HBV	68 (75.6)	30 (78.9)	38 (73.1)	0.522
Liver cirrhosis	60 (66.7)	27 (71.1)	33 (63.5)	0.451
ECOG PS				0.331
0	35 (38.9)	17 (44.7)	18 (34.6)	
1	55 (61.1)	21 (55.3)	34 (65.4)	
Child-Pugh stage				0.301
Stage A	71 (78.9)	28 (73.7)	43 (82.7)	
Stage B	19 (21.1)	10 (26.3)	9 (17.3)	
Maximum tumor diameter (cm)	9.2 (6.0–11.0)	9.5 (6.9–11.0)	8.8 (5.4–11.0)	0.202
Maximum tumor diameter ≥ 10 cm	33 (36.7)	15 (39.5)	18 (34.6)	0.637
Portal vein invasion	59 (65.6)	27 (71.1)	32 (61.5)	0.348
Extrahepatic disease	35 (38.9)	16 (42.1)	19 (36.5)	0.593
BCLC stage				
Stage C	90 (100.0)	38 (100.0)	52 (100.0)	1.000
CNLC stage				0.531
Stage IIb	19 (21.1)	7 (18.4)	12 (23.1)	
Stage IIIa	36 (40.0)	15 (39.5)	21 (40.4)	
Stage IIIb	35 (38.9)	16 (42.1)	19 (36.5)	
AFP (ng/mL)	314.9 (95.7-1878.4)	488.6 (110.1-2235.1)	250.6 (63.7-1512.5)	0.303
AFP ≥ 200 ng/mL	53 (58.9)	25 (65.8)	28 (53.8)	0.255
ALB (g/L)	38.7 (34.5–42.5)	37.3 (34.0-41.8)	39.1 (35.6–43.6)	0.259
Abnormal ALB	38 (42.7)	18 (48.6)	20 (38.5)	0.338
TBIL (µmol/L)	16.9 (12.0-23.9)	19.0 (12.9–29.9)	15.0 (11.0-19.9)	0.097
Abnormal TBIL	22 (24.7)	15 (40.5)	7 (13.5)	0.004
ALT (U/L)	28.0 (19.2–44.0)	27.0 (19.0–44.0)	28.0 (19.6–44.3)	0.777
Abnormal ALT	21 (23.6)	10 (27.0)	11 (21.2)	0.520
AST (U/L)	40.0 (26.0-60.5)	37.5 (25.3–61.5)	40.5 (26.3–60.5)	0.587
Abnormal AST	40 (45.5)	17 (47.2)	23 (44.2)	0.782
ALP (U/L)	117.0 (83.0-166.0)	114.0 (88.0-145.0)	127.5 (83.0-170.5)	0.326
Abnormal ALP	30 (34.5)	10 (28.6)	20 (38.5)	0.341
Times of TACE	1.6 ± 0.6	1.7 ± 0.5	1.5 ± 0.6	0.156

HCC, hepatocellular carcinoma; IA-TACE, immune checkpoint inhibitors plus apatinib combined with transcatheter chemoembolization; A-TACE, apatinib combined with transcatheter chemoembolization; HBV, hepatitis B virus; ECOG PS, Eastern Cooperative Oncology Group performance status; BCLC, Barcelona Clinic Liver Cancer; CNLC, China liver cancer; AFP, alpha-fetoprotein; ALB, albumin; TBIL, total bilirubin; ALT, alanine aminotransferase; AST, aspartate aminotransferase; ALP, alkaline phosphatase

Data were presented by median (interquartile range), number (percentage), and mean ± standard deviation. *P* value was determined by comparing HCC patients with A-TACE and with IA-TACE.

### Treatment response

The proportions of patients with complete response (CR), partial response (PR), stable disease (SD), and progressive disease (PD) were 13.2%, 44.7%, 28.9%, and 13.2%, respectively in IA-TACE group; while they were 9.6%, 26.9%, 40.4%, and 23.1%, respectively in A-TACE group. Comparison between groups revealed that the best response was better in IA-TACE group compared with A-TACE group, but did not reach statistical significance (*P* = 0.063). The objective response rate (ORR) was also higher in IA-TACE group compared with A-TACE group, although no statistical significance was observed (57.9% vs. 36.5%, *P* = 0.055). Disease control rate (DCR) was not different between the two groups (86.8% vs. 76.9%, *P* = 0.248) (Table 2).

**Table 2:** The best response of advanced HCC patients

Items	All (*N* = 90)	IA-TACE (*n* = 38)	A-TACE (*n* = 52)	*P* value
Best response				0.063
CR	10 (11.1)	5 (13.2)	5 (9.6)	
PR	31 (34.4)	17 (44.7)	14 (26.9)	
SD	32 (35.6)	11 (28.9)	21 (40.4)	
PD	17 (18.9)	5 (13.2)	12 (23.1)	
ORR	41 (45.6)	22 (57.9)	19 (36.5)	0.055
DCR	73 (81.1)	33 (86.8)	40 (76.9)	0.284

HCC, hepatocellular carcinoma; IA-TACE, immune checkpoint inhibitors plus apatinib combined with transcatheter chemoembolization; A-TACE, apatinib combined with transcatheter chemoembolization; CR, complete response; PR, partial response; SD, stable disease; PD, progressive disease; ORR, objective response rate; DCR, disease control rate

Data were presented by number (percentage). *P* value was determined by comparing HCC patients with A-TACE and with IA-TACE

Treatment response was also compared in IA-TACE group among patients with different types of ICIs. It was shown that best response, ORR, and DCR were not different among patients with different types of ICIs (all *P* > 0.05) (Supplementary Table 1).

### PFS and OS

**Fig. 1:**
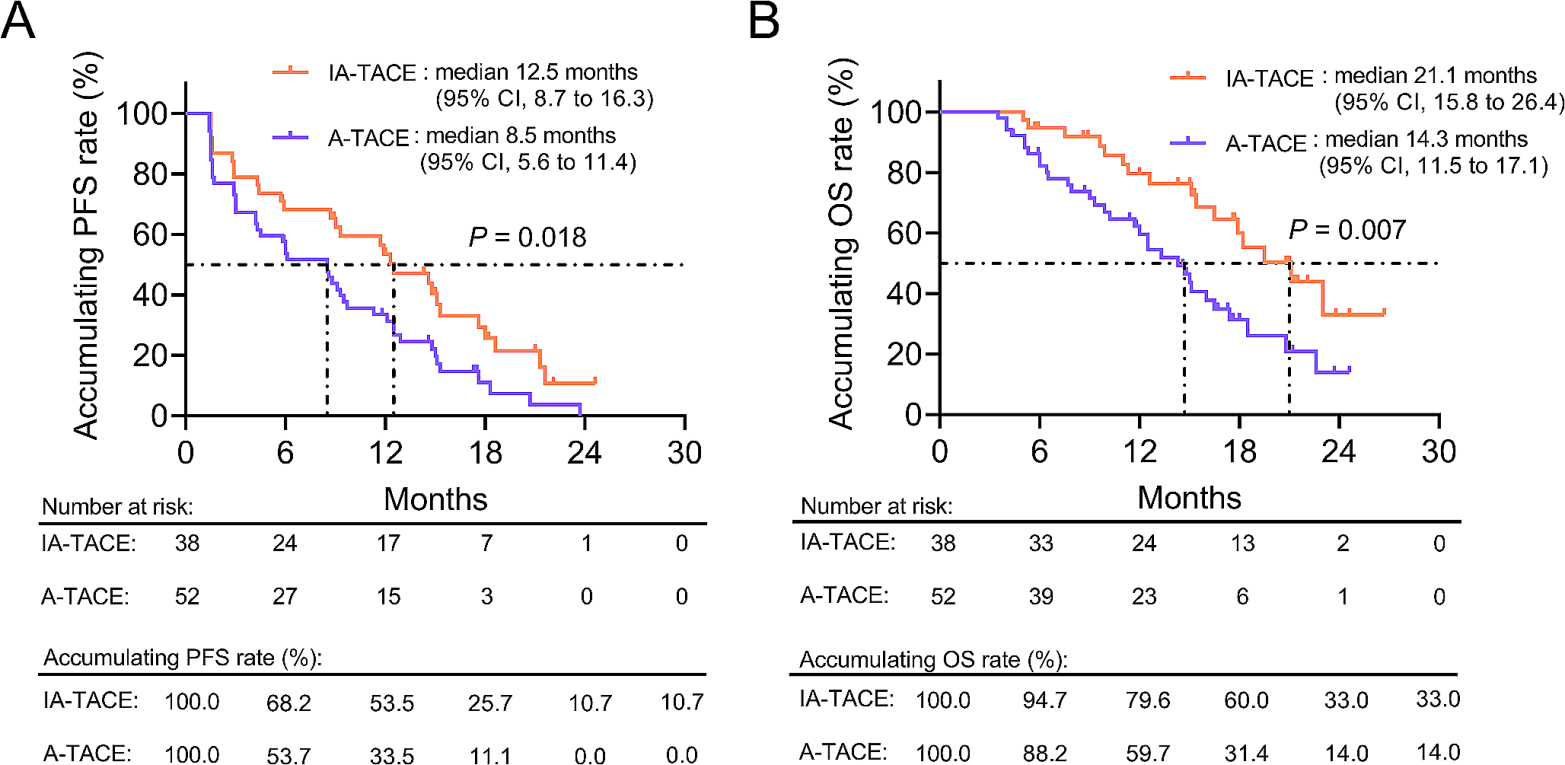
Survival between IA-TACE and A-TACE groups. Comparison of PFS (**A**) and OS (**B**) between IA-TACE group and A-TACE groups

The median (95% confidence interval (CI)) PFS was 12.5 (8.7–16.3) months in IA-TACE group and 8.5 (5.6–11.4) months in A-TACE group. The Log-rank test revealed that accumulating PFS was improved in IA-TACE group compared with A-TACE group (*P* = 0.018) (Fig. 1A). The median (95% CI) OS was 21.1 (15.8–26.4) months in IA-TACE group and 14.3 (11.5–17.1) months in A-TACE group. Similarly, accumulating OS was also prolonged in IA-TACE group compared with A-TACE group (*P* = 0.007) (Fig. 1B).

In IA-TACE group, PFS and OS were also compared between patients who received PD-1 inhibitors and those who received PD-L1 inhibitors. Accumulating PFS was better in patients who received PD-1 inhibitors compared those who received PD-L1 inhibitors (*P* = 0.027) (Supplementary Fig. 1A), but OS was not different between these two types of patients (*P* = 0.282) (Supplementary Fig. 1B).

### Factors affecting PFS and OS

Univariate Cox regression model showed that IA-TACE (*P* = 0.022) and times of TACE (*P* < 0.001) were associated with prolonged PFS. Maximum tumor diameter ≥ 10 cm (*P* = 0.005), portal vein invasion (*P* = 0.032), extrahepatic disease (*P* = 0.011), AFP ≥ 200 ng/mL (*P* = 0.024), and abnormal ALP (*P* = 0.035) were associated with worse PFS. By multivariate Cox regression model, IA-TACE (hazard ratio (HR) = 0.539, *P* = 0.038) and times of TACE (HR = 0.345, *P* < 0.001) were independently associated with improved PFS; whereas male sex (HR = 2.154, *P* = 0.046) and extrahepatic disease (HR = 2.473, *P* = 0.002) were independently associated with unfavorable PFS (Table 3).

**Table 3:** Univariate and multivariate Cox regression models for PFS

Characteristics	HR (95% CI)	*P* value
**Univariate model**		
IA-TACE	0.571 (0.354–0.921)	0.022
Age ≥ 60 years	1.375 (0.848–2.230)	0.197
Male sex	1.266 (0.662–2.421)	0.475
HBV	1.172 (0.679–2.022)	0.569
Liver cirrhosis	1.253 (0.768–2.045)	0.366
ECOG PS	1.393 (0.865–2.241)	0.172
Child-Pugh stage B	1.542 (0.883–2.695)	0.128
Maximum tumor diameter ≥ 10 cm	1.987 (1.235–3.198)	0.005
Portal vein invasion	1.710 (1.047–2.792)	0.032
Extrahepatic disease	1.836 (1.148–2.935)	0.011
AFP ≥ 200 ng/mL	1.735 (1.074–2.802)	0.024
Abnormal ALB	1.205 (0.759–1.912)	0.429
Abnormal TBIL	1.183 (0.699–2.002)	0.530
Abnormal ALT	0.847 (0.491–1.462)	0.552
Abnormal AST	0.936 (0.584–1.501)	0.785
Abnormal ALP	1.763 (1.040–2.991)	0.035
Times of TACE	0.303 (0.187–0.489)	< 0.001
**Multivariate model (backward)**		
IA-TACE	0.539 (0.300-0.967)	0.038
Male sex	2.154 (1.014–4.575)	0.046
Maximum tumor diameter ≥ 10 cm	1.603 (0.976–2.631)	0.062
Extrahepatic disease	2.473 (1.404–4.356)	0.002
Times of TACE	0.345 (0.209–0.570)	< 0.001

PFS, progression-free survival; HR, hazard ratio; CI, confidence interval; IA-TACE, immune checkpoint inhibitors plus apatinib combined with transcatheter chemoembolization; HBV, hepatitis B virus; ECOG PS, Eastern Cooperative Oncology Group performance status; AFP, alpha-fetoprotein; ALB, albumin; TBIL, total bilirubin; ALT, alanine aminotransferase; AST, aspartate aminotransferase; ALP, alkaline phosphatase

IA-TACE (*P* = 0.009) and times of TACE (*P* < 0.001) were associated with improved OS, while maximum tumor diameter ≥ 10 cm (*P* = 0.018), portal vein invasion (*P* = 0.028), and extrahepatic disease (*P* = 0.043) were associated with shorter OS. The multivariate Cox regression model showed that IA-TACE (HR = 0.447, *P* = 0.025) and times of TACE (HR = 0.244, *P* < 0.001) were independently associated with longer OS. Extrahepatic disease (HR = 2.615, *P* = 0.005) was independently associated with worse OS (Table 4).

**Table 4:** Univariate and multivariate Cox regression models for OS

Characteristics	HR (95% CI)	*P* value
**Univariate model**		
IA-TACE	0.446 (0.243–0.818)	0.009
Age ≥ 60 years	1.603 (0.876–2.933)	0.126
Male sex	1.414 (0.599–3.335)	0.429
HBV	1.392 (0.674–2.885)	0.370
Liver cirrhosis	1.425 (0.751–2.701)	0.278
ECOG PS	1.529 (0.830–2.819)	0.173
Child-Pugh stage B	1.142 (0.548–2.381)	0.723
Maximum tumor diameter ≥ 10 cm	1.991 (1.124–3.525)	0.018
Portal vein invasion	2.027 (1.081–3.804)	0.028
Extrahepatic disease	1.814 (1.020–3.224)	0.043
AFP ≥ 200 ng/mL	1.510 (0.834–2.736)	0.174
Abnormal ALB	0.989 (0.554–1.766)	0.970
Abnormal TBIL	1.199 (0.620–2.317)	0.590
Abnormal ALT	0.654 (0.316–1.352)	0.252
Abnormal AST	0.678 (0.373–1.231)	0.202
Abnormal ALP	1.432 (0.760–2.699)	0.267
Times of TACE	0.193 (0.103–0.361)	< 0.001
**Multivariate model (backward)**		
IA-TACE	0.447 (0.221–0.905)	0.025
Male sex	2.565 (0.978–6.727)	0.056
Extrahepatic disease	2.615 (1.338–5.109)	0.005
Times of TACE	0.244 (0.131–0.457)	< 0.001

OS, overall survival; HR, hazard ratio; CI, confidence interval; IA-TACE, immune checkpoint inhibitors plus apatinib combined with transcatheter chemoembolization; HBV, hepatitis B virus; ECOG PS, Eastern Cooperative Oncology Group performance status; AFP, alpha-fetoprotein; ALB, albumin; TBIL, total bilirubin; ALT, alanine aminotransferase; AST, aspartate aminotransferase; ALP, alkaline phosphatase

**Table 5:** Adverse events

Events	All (*N* = 90)	IA-TACE (*n* = 38)	A-TACE (*n* = 52)	*P* value
Pain	52 (57.8)	20 (52.6)	32 (61.5)	0.398
Fever	39 (43.3)	18 (47.4)	21 (40.4)	0.509
Hand-foot syndrome	38 (42.2)	15 (39.5)	23 (44.2)	0.652
Hypertension	35 (38.9)	16 (42.1)	19 (36.5)	0.593
Nausea and vomiting	26 (28.9)	12 (31.6)	14 (26.9)	0.630
Fatigue	23 (25.6)	8 (21.1)	15 (28.8)	0.402
Leukopenia	21 (23.3)	11 (28.9)	10 (19.2)	0.282
Rash	19 (21.1)	10 (26.3)	9 (17.3)	0.301
Anemia	18 (20.0)	9 (23.7)	9 (17.3)	0.455
Diarrhea	15 (16.7)	7 (18.4)	8 (15.4)	0.703
Thrombocytopenia	15 (16.7)	7 (18.4)	8 (15.4)	0.703
Neutropenia	13 (14.4)	7 (18.4)	6 (11.5)	0.359
Thyroid dysfunction	8 (8.9)	6 (15.8)	2 (3.8)	0.066
RCCEP	4 (4.4)	4 (10.5)	0 (0.0)	0.029

IA-TACE, immune checkpoint inhibitors plus apatinib combined with transcatheter chemoembolization; A-TACE, apatinib combined with transcatheter chemoembolization; RCCEP, reactive cutaneous capillary endothelial proliferation

*P* value was determined by comparing HCC patients with A-TACE and with IA-TACE

### Subgroup analyses of PFS and OS

**Fig. 2:**
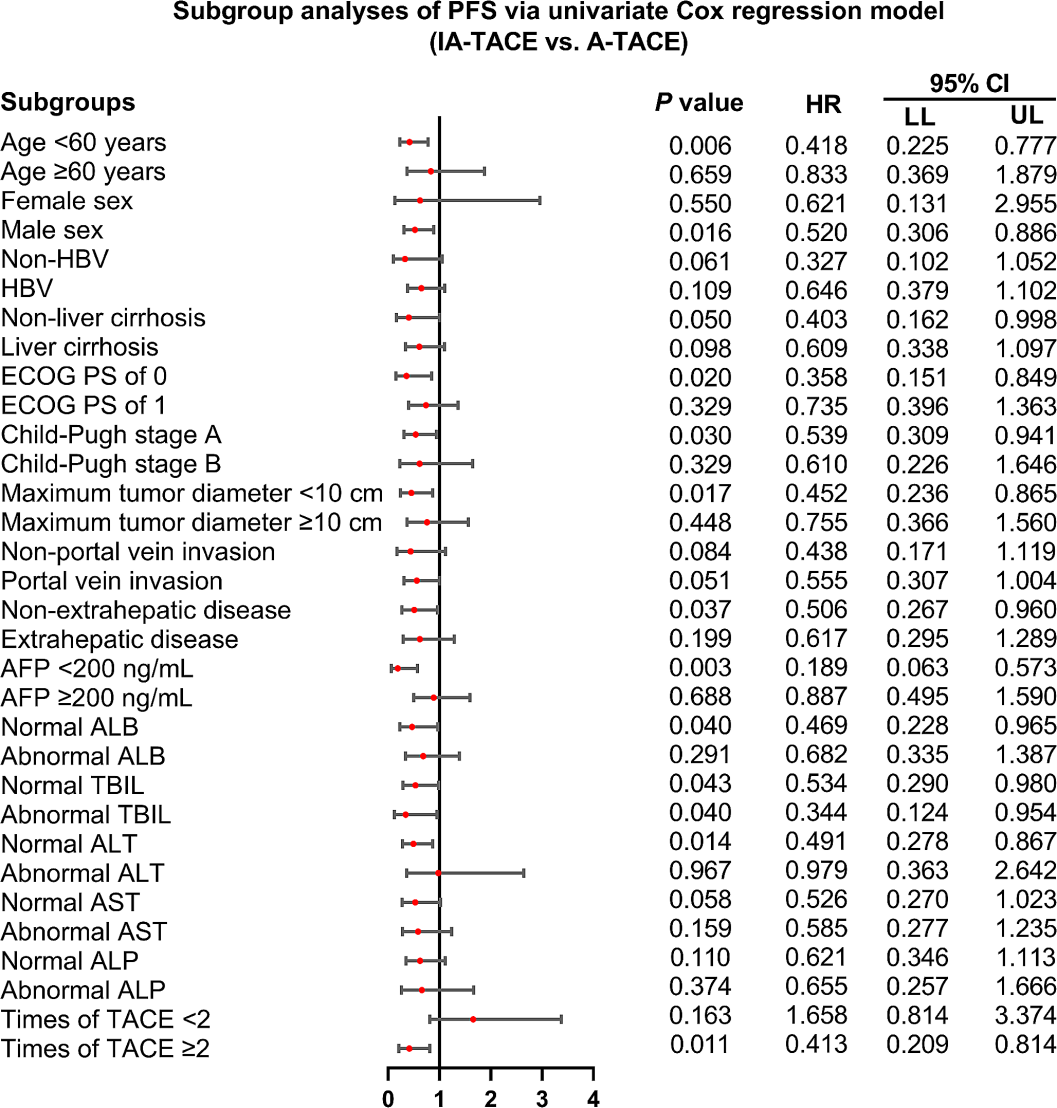
Subgroup analyses of PFS

IA-TACE was associated with prolonged PFS in subgroups of patients with age < 60 years (*P* = 0.006), males (*P* = 0.016), patients without liver cirrhosis (*P* = 0.050), patients with ECOG PS of 0 (*P* = 0.020), patients with Child-Pugh stage A (*P* = 0.030), patients with maximum tumor diameter < 10 cm (*P* = 0.017), patients without extrahepatic disease (*P* = 0.037), patients with AFP < 200 ng/mL (*P* = 0.003), patients with normal ALB (*P* = 0.040), patients with normal TBIL (*P* = 0.043), patients with abnormal TBIL (*P* = 0.040), patients with normal ALT (*P* = 0.014), and patients with times of TACE ≥ 2 (*P* = 0.011) (Fig. 2). While in other subgroups, IA-TACE (vs. A-TACE) could not provide benefit in PFS.

**Fig. 3:**
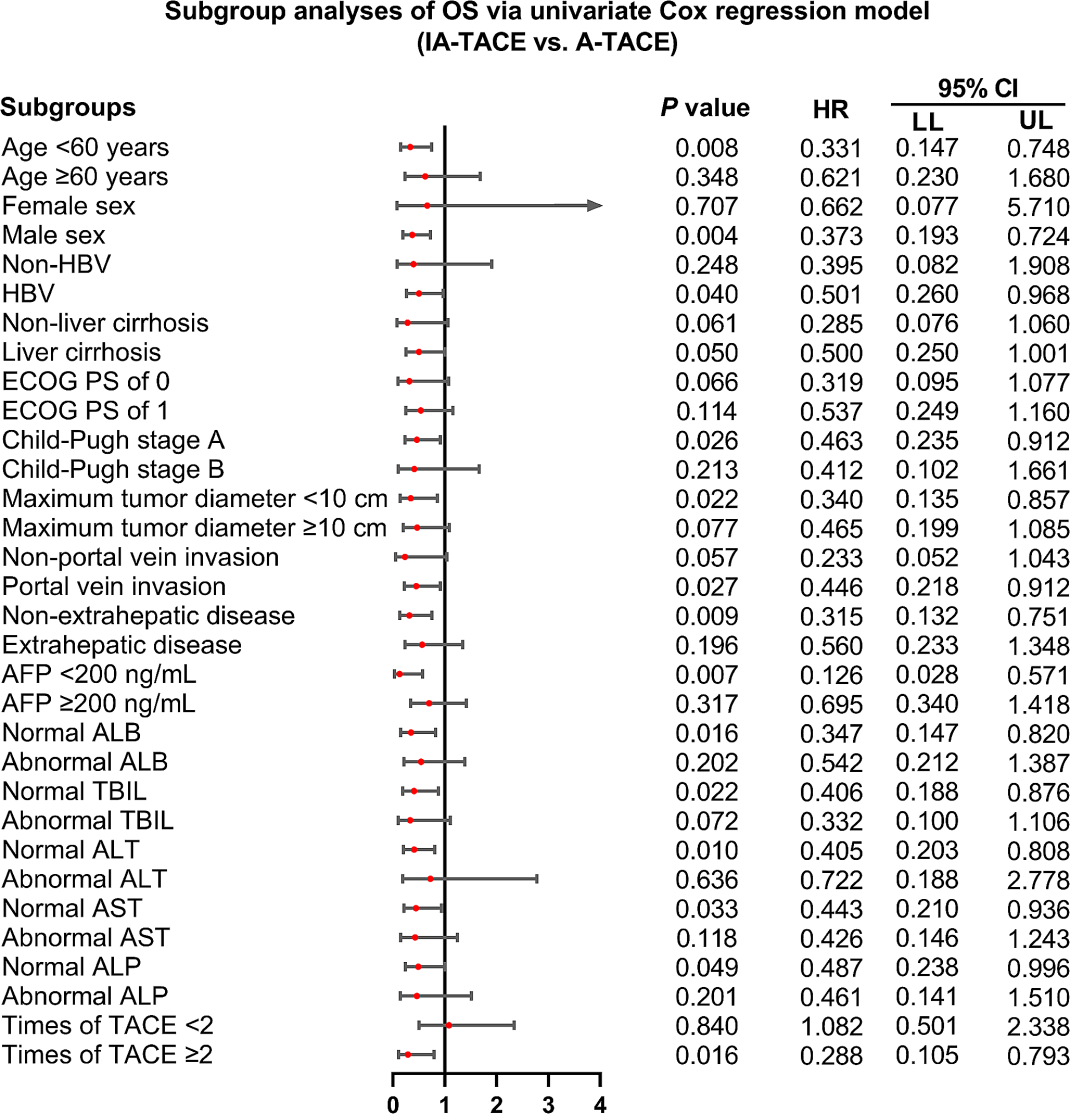
Subgroup analyses of OS

IA-TACE was associated with improved OS in subgroups of patients with age < 60 years (*P* = 0.008), males (*P* = 0.004), patients with HBV infection (*P* = 0.040), patients with Child-Pugh stage A (*P* = 0.026), patients with maximum tumor diameter < 10 cm (*P* = 0.022), patients with portal vein invasion (*P* = 0.027), patients without extrahepatic disease (*P* = 0.009), patients with AFP < 200 ng/mL (*P* = 0.007), patients with normal ALB (*P* = 0.016), patients with normal TBIL (*P* = 0.022), patients with normal ALT (*P* = 0.010), patients with normal AST (*P* = 0.033), patients with normal ALP (*P* = 0.049), and patients with times of TACE ≥ 2 (*P* = 0.016) (Fig. 3). While in other subgroups, IA-TACE (vs. A-TACE) could not provide benefit in OS.

### Safety profile

The most commonly occurring adverse events in the IA-TACE group were pain (52.6%), fever (47.4%), hypertension (42.1%), and hand-foot syndrome (39.5%). While the most commonly occurring adverse events in A-TACE group were pain (61.5%), hand-foot syndrome (44.2%), fever (40.4%), and hypertension (36.5%). Comparison analyses revealed that only the incidence of reactive cutaneous capillary endothelial proliferation (RCCEP) was higher in IA-TACE group compared with A-TACE group (10.5% vs. 0.0%, *P* = 0.029), but the incidences of other adverse events were not different between the two groups.

## Discussion

TACE exerts anti-tumor effects through combining the blockage of tumor-feeding arteries using microparticles or microspheres and locoregional chemotherapeutic agents (Hatanaka et al. 2023). However, the blockage of tumor-feeding artery would induce hypoxia in the tumor, which would promote the secretion of VEGF to enhance angiogenesis, thus inducing tumor progression and metastasis (Rhee et al. 2016). When TACE is combined with apatinib, the VEGF receptor signaling would be inhibited, subsequently suppressing the progression and metastasis of the tumor, which could yield a promoted treatment efficacy (Liu J, Xu, et al. 2020). Regarding the rationale for combining apatinib with ICIs, it is reported that VEGF could induce the exhaustion of CD8^+^ cytotoxic T cells to suppress anti-tumor immunity (Kim et al. 2019). Therefore, apatinib could provide a better immune microenvironment for ICIs, thus promoting the treatment efficacy of ICIs. In terms of TACE and ICIs, previous studies report that TACE could induce immunogenic cell death, thus providing a more favorable immune microenvironment for ICIs (Chang et al. 2019). Based on these theories, several studies have explored the efficacy of TACE combined with apatinib and ICIs in patients with advanced HCC (Zhu et al. 2022; Duan et al. 2023; Liu et al. 2023; Xia et al. 2023). These studies report that the ORR in patients with advanced HCC who receive TACE combined with apatinib and ICIs ranges from 43.2 to 53.6%, and the DCR ranges from 67.6 to 88.4%. Our study revealed that in IA-TACE group, the ORR was 57.9% and the DCR was 86.8%. These data were similar to those of previous studies (Zhu et al. 2022; Duan et al. 2023; Liu et al. 2023; Xia et al. 2023). In addition, our study also found that the treatment response was numerically higher in IA-TACE group compared with A-TACE group, but did not reach statistical significance. The possible explanations were: (1) as mentioned above, TACE, ICIs, and apatinib, either two of them showed synergistic anti-tumor effects (Chang et al. 2019; Kim et al. 2019; Liu J, Xu, et al. 2020), which resulted in a better treatment efficacy compared with TACE combined with apatinib; (2) the sample size of this study was relatively small, which might restrict the statistical power.

The overall survival of patients with advanced HCC is still unfavorable and searching for potential treatments with survival benefits in these patients is urgent (Elderkin et al. 2023). Previous studies report a median PFS of 6.9 to 14.0 months and a median OS of 15.4 to 24.5 months in advanced HCC who receive TACE combined with apatinib and ICIs (Zhu et al. 2022; Duan et al. 2023; Liu et al. 2023; Sun et al. 2023; Xia et al. 2023). The current study showed that the median PFS was 12.5 months (95% CI: 8.7 to 16.3 months) and the median OS was 21.1 months (95% CI: 15.8 to 26.4 months) in IA-TACE group, which was in accordance with these previous studies (Zhu et al. 2022; Duan et al. 2023; Liu et al. 2023; Sun et al. 2023; Xia et al. 2023). Meanwhile, our study also revealed that PFS and OS were both improved in IA-TACE group compared with A-TACE group, which was also in line with previous studies (Zhu et al. 2022; Duan et al. 2023; Liu et al. 2023; Sun et al. 2023; Xia et al. 2023). Moreover, IA-TACE (vs. A-TACE) was independently associated with better PFS and OS. These findings suggested the survival benefit of TACE combined with apatinib and ICIs for patients with advanced HCC. The explanation for these findings was that as mentioned above, TACE combined with apatinib and ICIs yielded a better treatment efficacy, which directly resulted in a more favorable survival outcome. The multivariate Cox regression model also revealed that male sex was independently associated with shorter PFS. The possible explanation was that the sample size of this study was small, and the predominant of patients in the current study were males; therefore, the occasional cases may affect the results of multivariate Cox regression models. It was also found that times of TACE was independently associated with longer PFS and OS, which was similar to the findings of a previous study (Wang et al. 2023). It should be clarified that the multivariate Cox regression model could not clarify the causality between dependent and independent variables. It is more likely that patients with longer PFS and OS could receive more times of TACE.

The safety of anti-tumor agents is noteworthy since patients with cancers are unable to benefit from them if intolerance occurs. The most commonly occurring adverse events of TACE, apatinib, and ICIs are fever, abdominal pain, hypertension, hand-foot syndrome, and nausea and vomiting (Geng et al. 2018; Lawson et al. 2023; Curkovic et al. 2024). According to previous studies, TACE combined with apatinib and ICIs shows comparable safety with TACE combined with apatinib (Zhu et al. 2022; Duan et al. 2023; Liu et al. 2023; Sun et al. 2023; Xia et al. 2023). Our study also revealed similar findings, that the incidences of most adverse events were comparable between groups. Only the incidence of RCCEP was higher in IA-TACE group compared with A-TACE group (10.5% vs. 0.0%). RCCEP is a common immune-related adverse event of camrelizumab (Chen et al. 2020a, b). It is associated with the vascular neogenesis effect of camrelizumab, which is mild and manageable (Chen et al. 2020a, b).

Several limitations of this study should be mentioned. First, the retrospective design of this study might induce bias in the results, and the findings of this study should be verified by randomized, controlled trials. Second, the sample size of this study was not large enough, and further large-scale studies should be conducted. Third, this study only enrolled patients with advanced HCC; thus, the findings of this study might not be applicable in patients with intermediate stage HCC but not able or unwilling for tumor resection. Fourth, the activity of HBV or status of hepatitis C virus (HCV) was not available, and thus the association of HBV activity and HCV with treatment response should be further investigated.

## Conclusion

In conclusion, TACE combined with apatinib and ICIs yields more favorable survival and comparable safety compared with TACE combined with apatinib in patients with advanced HCC. The findings of this study propose the possibility of TACE combined with apatinib and ICIs for the treatment of advanced HCC. Further large-scale studies and randomized, controlled trials should be conducted to compare the efficacy and safety of TACE combined with apatinib and ICIs versus TACE combined with apatinib.

## Electronic supplementary material

Below is the link to the electronic supplementary material.

Supplementary Material 1

## Data Availability

The datasets generated during and/or analysed during the current study are available from the corresponding author on reasonable request.
