# Supplementary material for: Efficacy and safety of apatinib plus immune checkpoint inhibitors and transarterial chemoembolization for the treatment of advanced hepatocellular carcinoma
Source: J Cancer Res Clin Oncol. 2024 Jul 8;150(7):340. doi: 10.1007/s00432-024-05854-8 (PMC11230948; doi:10.1007/s00432-024-05854-8)

**Supplementary Table 1.** The best response of advanced HCC patients with different types of ICIs.

| Items | Camrelizumab  (n = 19) | Sintilimab  (n = 7) | Atezolizumab  (n = 9) | Tislelizumab  (n = 3) | *P* value |
| --- | --- | --- | --- | --- | --- |
| Best response |  |  |  |  | 0.119 |
| CR | 4 (21.1) | 0 (0.0) | 1 (11.1) | 0 (0.0) |  |
| PR | 11 (57.9) | 3 (42.9) | 2 (22.2) | 1 (33.3) |  |
| SD | 2 (10.5) | 3 (42.9) | 5 (55.6) | 1 (33.3) |  |
| PD | 2 (10.5) | 1 (14.3) | 1 (11.1) | 1 (33.3) |  |
| ORR | 15 (78.9) | 3 (42.9) | 3 (33.3) | 1 (33.3) | 0.053 |
| DCR | 17 (89.5) | 6 (85.7) | 8 (88.9) | 2 (66.7) | 0.654 |

HCC, hepatocellular carcinoma; ICIs, immune checkpoint inhibitors; CR, complete response; PR, partial response; SD, stable disease; PD, progressive disease; ORR, objective response rate; DCR, disease control rate.

Data were presented by number (percentage). *P* value was determined by comparing HCC patients with different administration of ICIs.

**Supplementary Figure 1.** Survival in IA-TACE group between PD-1 inhibitors and PD-L1 inhibitors. Comparison of PFS (**A**) and OS (**B**) between patients who received PD-1 inhibitors and those who received PD-L1 inhibitors.


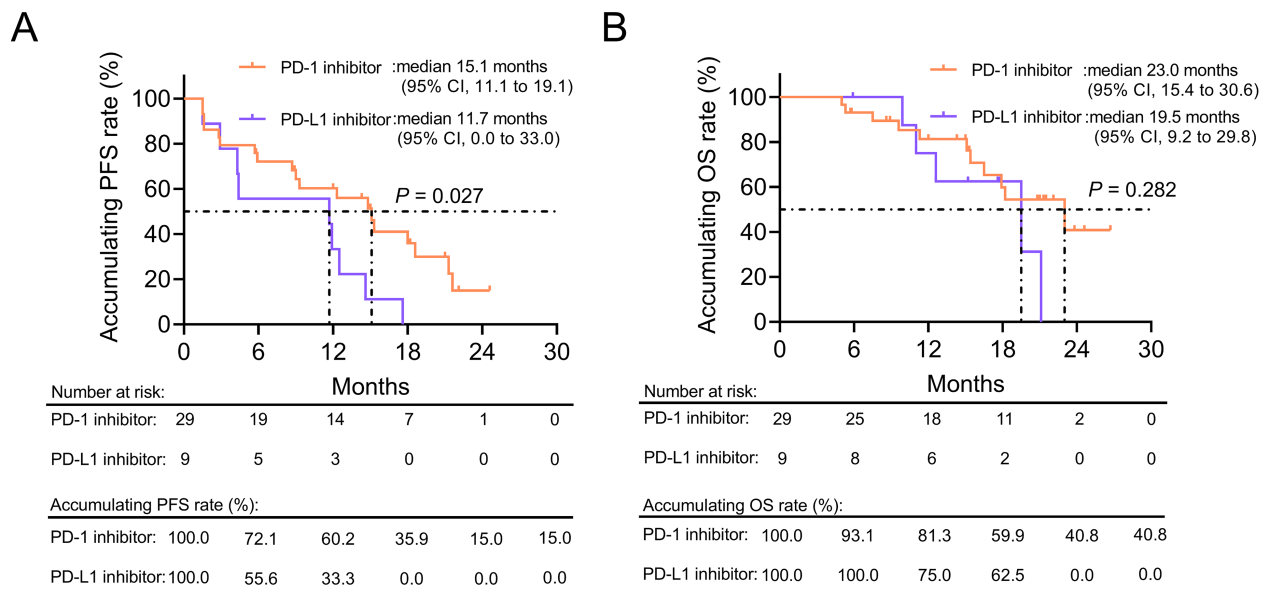

Supplement: Supplementary file 1 — Supplementary Material 1 [file 432_2024_5854_MOESM1_ESM.docx]
